# Photothermal Responsivity of van der Waals Material-Based Nanomechanical Resonators

**DOI:** 10.3390/nano12152675

**Published:** 2022-08-04

**Authors:** Myrron Albert Callera Aguila, Joshoua Condicion Esmenda, Jyh-Yang Wang, Yen-Chun Chen, Teik-Hui Lee, Chi-Yuan Yang, Kung-Hsuan Lin, Kuei-Shu Chang-Liao, Sergey Kafanov, Yuri A. Pashkin, Chii-Dong Chen

**Affiliations:** 1 Department of Engineering and System Science, National Tsing Hua University, Hsinchu 30013, Taiwan; 2 Nano Science and Technology Program, Taiwan International Graduate Program, Academia Sinica, Taipei 11529, Taiwan; 3 Institute of Physics, Academia Sinica, Nankang, Taipei 11529, Taiwan; 4 Department of Physics, Lancaster University, Lancaster LA1 4YB, UK

**Keywords:** van der Waals materials, nanomechanical resonators, Fabry–Pérot interferometry, photothermal response, static displacement, absorptive heating, NbSe_2_ drumheads

## Abstract

Nanomechanical resonators made from van der Waals materials (vdW NMRs) provide a new tool for sensing absorbed laser power. The photothermal response of vdW NMRs, quantified from the resonant frequency shifts induced by optical absorption, is enhanced when incorporated in a Fabry–Pérot (FP) interferometer. Along with the enhancement comes the dependence of the photothermal response on NMR displacement, which lacks investigation. Here, we address the knowledge gap by studying electromotively driven niobium diselenide drumheads fabricated on highly reflective substrates. We use a FP-mediated absorptive heating model to explain the measured variations of the photothermal response. The model predicts a higher magnitude and tuning range of photothermal responses on few-layer and monolayer NbSe_2_ drumheads, which outperform other clamped vdW drum-type NMRs at a laser wavelength of 532 nm. Further analysis of the model shows that both the magnitude and tuning range of NbSe_2_ drumheads scale with thickness, establishing a displacement-based framework for building bolometers using FP-mediated vdW NMRs.

## 1. Introduction

Nanomechanical resonators (NMRs) embedded in an optical cavity are valuable platforms for studying weak forces due to the enhanced coupling between light and motion [1]. Enhanced coupling improves the capability of nanomechanical resonators to demonstrate nonlinear dynamics [2] and sense heat transport in suspended nanostructures [3]. Resonators interacting with optical elements enjoy additional degree of spatiality [4,5,6,7,8], extremely large optomechanical coupling at ambient temperature [9,10], and reduced mode volume due to breakthrough technologies in focusing laser beams via free space optics [11,12,13], fiber optics [14,15,16] and near-field interactions with multiplexed on-chip optical waveguides and tapered fibre [17,18].

Heating induced by the probe laser remains a concern in the optical readout of NMRs [19,20]. Photothermal effects [3,15,21,22] emerge once the laser illuminates a region of the resonator and raises the temperature of the mechanical mode [20], while the process hinders ground-state cooling of the mechanical mode [23], it enables sensing of incident laser power with the aid of on-chip Fabry–Pérot (FP) cavities. NMRs fabricated with suspended van der Waals (vdW) materials [24,25,26] show promising photothermal sensitivities [27] due to reduced mass, and layer-dependent mechanical, optical, and thermal properties. Few-layer and multilayer niobium diselenide (NbSe_2_) is a candidate vdW material for low-noise, highly responsive photodetectors at ultraviolet [28] and infrared wavelengths [29,30].

There are several approaches to quantifying the power absorbed by vdW NMRs. One can measure the mechanical mode temperature by resolving thermomechanical motion [6,31] while varying the incident laser power. Such detection requires low mass and high quality factors of NMRs, which can be difficult to ensure for vdW materials [24,25,26]. A less stringent yet popular method involves monitoring of the resonant frequency shift of an electromotively driven vdW NMRs [3,27], and following its dependence on the incident laser power. In both approaches, light absorption depends not only on the FP structure and wavelength, but also on the resonator displacement from the initial equilibrium position. Understanding the displacement-dependent absorptive response may provide insights into managing FP-mediated heat flow in NMRs, while experiments on NMRs fabricated from various types of vdW materials have been published, photothermal sensing with NbSe_2_ NMRs has not yet been reported. NbSe_2_ has low thermal conductivity [32], is flexible [33] and has large fracture strain [34]. This combination of properties enables the realization of low power, photothermal-strain-sensitive detectors.

In this paper, we explore the influence of the resonator displacement on the sensitivity of the FP-based vdW NMRs to incident light by investigating the photothermal response of electromotively driven NbSe_2_ drumheads. We propose an FP-mediated absorptive heating model that accounts for resonator displacement to explain the observed variations in the measured photothermal response. The model consequently reveals a large tuning range of photothermal responsivity as the drumhead moves towards the bottom electrodes. We extend the model to drumheads of varying thicknesses and materials to gain insight into the geometric and material impact of FP-mediated heating on NMRs.

## 2. Materials and Methods

### 2.1. Device Design and Fabrication

**Figure 1:**
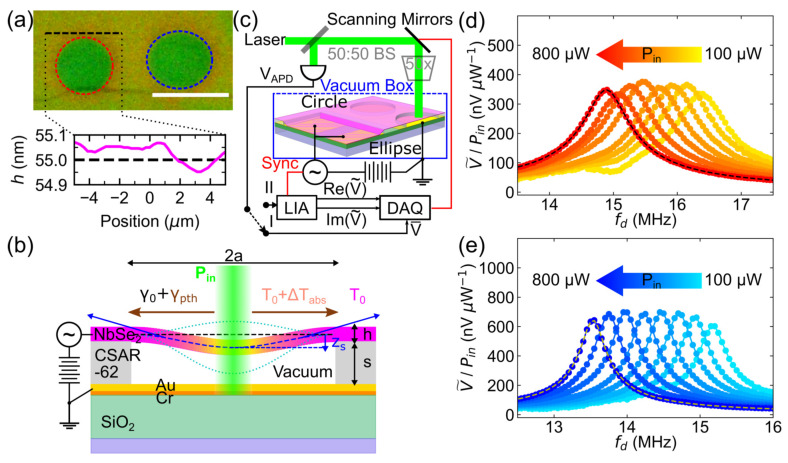
Measurement setup and device. (**a**) Optical micrograph of the circular (red dashed circle) and elliptical (blue dashed ellipse) drumhead resonators under study. The white scale bar corresponds to a length of 10 μm. The black dashed line on the micrograph represents the average thickness of the NbSe_2_ flake as measured by the Multilayer Interference Approach. (**b**) Schematic diagram of laser-induced photothermal heating of an electromotively driven drumhead resonator. Cross-section showing the net tension exerted by the drumhead resonators under photothermal heating at the middle of the drumhead. (**c**) Optical interferometric setup used to track the mechanical frequency of multilayered NbSe_2_ flake mechanical resonators. Measured driven mechanical responses of circular and elliptical drumhead resonators at increasing incident laser power are shown in (**d**,**e**), respectively. The data are represented with dot markers connected with lines. The devices are electromotively driven at $V_{DC}=4\;$V and $V_{AC}=0.125\;$V${}_{pk}$. The rightmost colored responses in (**d**,**e**) represent the driven responses resolved at the lowest incident powers $P_{in}$. The consecutive responses darken with increasing $P_{in}$. Dashed lines refer to the driven resonator fits.

Figure 1a shows the optical micrograph of devices A (circular) and B (elliptical). Bulk NbSe_2_ flakes bought from HQ Graphene are mechanically exfoliated with PDMS stamps [8,35,36,37]. The flakes are then transferred on a lithographically patterned Au-Cr-SiO_2_-Si substrate covered with electron-beam patterned resist CSAR-62 via dry deterministic transfer [8,36,37,38]. The freestanding regions of the flake above the patterned drum holes of the resist represent the drumhead resonator devices. Device A has a radius $a_{A}=b_{A}=3.5\;\mu$m and device B has a major radius $a_{B}=4.0\;\mu$m and a minor radius $b_{B}=3.5\;\mu$m. The cross-section layout of the FP structure is shown in Figure 1b. The 40 nm-thick Au and 20 nm-thick Cr electrodes are deposited on 543 nm-thick SiO_2_ to act as electrical ground and mirror. The CSAR-62 thickness *s* determines the designed FP cavity length.

### 2.2. Electromotive Actuation and Optical Detection Scheme

The mechanical drums are set into motion by an electromotive force originating from a combination of DC and oscillating voltages and as shown in Figure 1b,c. The electromotive force can be written as [2]

(1) $$F_{em}=\frac{1}{2}\frac{dC_{m}}{dz}{\left[V_{DC}+V_{AC}\cos\left(2\pi f_{d}t\right)\right]}^{2},$$

where $C_{m}$ is the vacuum capacitance between the freestanding drumhead and the bottom electrode with distance defined by *s*, $V_{DC}$ is the DC voltage, $V_{AC}$ is the amplitude of the oscillating voltage at driving frequency $f_{d}$, *t* is time, and *z* is the out-of-plane (z-direction) displacement from the equilibrium position. The relation between the voltages and the time-dependent displacement for a tensioned electrostatic drum plate can be written in the form [8,39]

(2) $$\rho h\frac{\partial^{2}z\left(t\right)}{\partial t^{2}}+D_{P}\nabla^{4}z\left(t\right)-\gamma \nabla^{2}z\left(t\right)=\frac{F_{em}}{\pi a_{eff}^{2}},$$

where ρ is the mass density, *h* is the drumhead thickness, $a_{eff}$ is the effective radius of the drumhead, γ is the tension, and $D_{P}$ is the flexural rigidity of a circular plate. Equation (2) is the general equation that determines the vibrational amplitude for electromotively-driven tensioned drum plates.

The motion is detected through the optical interferometric detection scheme at a laser wavelength λ=532 nm, as shown in Figure 1c. The chip containing NMR drumheads is placed in a vacuum box with optical and electrical access and a base pressure of $6\;\times \;10^{-7}$ mBar. The laser beam, passing through a 50× objective, has a focused spot diameter of 1.9 μm and allows for spatial resolution of picometer vibrational amplitudes given the designed FP cavity length s=295 ± 10  nm as measured by a commercial stylus profilometer [8,36]. Scanning mirrors help align the spot of the probe laser beam at different positions on the drumhead. The reflected interfering light obtains redirected by the beamsplitter (50:50 BS) to the photodetector (PD). The data acquisition (DAQ) unit and the lock-in amplifier (LIA) read the PD output DC voltage $\bar{V}$ and AC voltage $\tilde{V}\left(f_{d}\right)$, respectively. By using the multilayer interface approach (MIA) [36,40], the spacer height is determined from the measured $\bar{V}$ to be s=296 nm, and the drumhead thickness h=55 nm (shown in Figure 1a), which is about 92 layers assuming a single layer thickness of 0.6nm [35]. The LIA measures $\tilde{V}\left(f_{d}\right)$ using the homodyne detection technique, with a time constant of 0.3 s and a time delay of 6 s to lock the phase at $f_{d}$ and measure the steady-state response of the NMR.

### 2.3. Photothermal Effect

When a laser beam illuminates a region of the drumhead, the drum surface reflects a fraction of the incident laser power $P_{in}$ for detection and absorbs some of the power accumulated in the FP cavity. The drumhead experiences radiation pressure and photothermal effects. Radiation pressure is exerted on the drumhead surface due to the momentum transfer between the drumhead and photons. This pressure is enhanced when incorporated into an FP structure due to multiple round trips of photons that are trapped between the drumhead and the bottom mirror. The tension generated from radiation pressure depends on $P_{in}$, λ and the reflectance of the drumhead and the reflective mirror in the FP structure [15,21]. The calculations in the Supplementary Materials show small induced tension for the highest applied $P_{in}$, indicating that the observed mechanical shift originates from the photothermal effect.

The photothermal effect for most van der Waals structure relies solely on laser-induced absorption [3]. The absorbed power can be described as $P_{abs}=P_{in}\chi A_{FP}$, where $A_{FP}$ is the total absorbance of the FP cavity and χ is the power fraction absorbed by the drumhead in the FP stack. The absorbance of the FP cavity for a given drumhead thickness *h* depends on the difference between the spacer height *s* and the resonator static displacement $z_{s}$. $P_{abs}$ heats the illuminated spot, and produces a temperature gradient due to the radial heat transfer to the drum clamps, as shown in Figure 1b. Consequently, the elevated temperature changes the mechanical tension exerted by the clamps by a photothermal tension, $\gamma_{pth}$. As illustrated in Figure 1b, $\gamma_{pth}$ is added to the existing initial mechanical tension $\gamma_{0}$ generated by the drum displacement.

### 2.4. Determination of Fabry–Pérot Absorbance via Multilayer Interference Approach

The absorbances of our multilayer stack are estimated by determining the reflectance and transmittance of the substrate used for our FP devices. For simplicity, the reflectance, transmittance, and absorbance calculations assume that the laser with a wavelength λ=532 nm originates from a point source in a vacuum and propagates towards the FP structure. Assuming that the thickness of the Si substrate is infinitely large, the reflection coefficient of the Au-Cr-SiO_2_-Si substrate acting as a bottom mirror is $\Gamma_{m}=0.665-0.565$j, numerically determined via MIA [36,41,42], and its corresponding reflectance is $R_{m}=0.761$. For an FP cavity with a vacuum spacer, we define the reflectance of the vacuum spacer to be [36]

(3) $$R_{FP}\left(h,z_{s}\right)={\left|\frac{r_{h}\left(1-e^{-2j\delta_{h}}\right)-\left(r_{h}^{2}-e^{-2j\delta_{h}}\right)\Gamma_{m}e^{-2j\delta_{s}}}{1-r_{1}^{2}e^{-2j\delta_{h}}-r_{h}\left(1-e^{-2j\delta_{h}}\right)\Gamma_{m}e^{-2j\delta_{s}}}\right|}^{2},$$

where $r_{h}=\left(1-{\hat{n}}_{res}\left(\lambda \right)\right)/\left(1+{\hat{n}}_{res}\left(\lambda \right)\right)$ is the reflection coefficient of the vacuum-flake interface, $\delta_{h}=2\pi {\hat{n}}_{res}\left(\lambda \right)h/\lambda$ is the optical phase thickness of the resonator with a complex-valued refractive index of the resonator material ${\hat{n}}_{res}$, and $\delta_{s}=2\pi {\hat{n}}_{vac}\left(s-z_{s}\right)/\lambda$ is the optical phase thickness of the vacuum spacer, with ${\hat{n}}_{vac}=1$.

Next, MIA is used to determine the substrate transmittance ($T_{sub}=0.03$) and absorbance ($A_{sub}=0.21$). Adding the nanomechanical resonator with a corresponding vacuum gap on the substrate leads to a vanishing transmittance of the overall FP stack $T_{FP}\approx 0$, thereby the absorbance of the FP-cavity system can be expressed as

(4) $$A_{FP}=A_{FP}\left(h,z_{s}\right)\approx 1-R_{FP}\left(h,z_{s}\right).$$

Combination of Equation (4) and Equation (3) provides the modeled absorbance $A_{FP}$ of the drumheads as a function of *h* and $z_{s}$.

Apart from $A_{FP}$, the proportion of light absorbed by the nanomechanical resonator in the FP stack, represented by χ, is determined using the TMM package in Python [42]. Laser radiation propagates towards the surface of the FP stack, where both forward (transmitted) and backward (reflected) electric field interfere at each interface. The resonator and the substrate, especially the metal electrodes, absorb some of the energy stored in the FP cavity while the vacuum does not. The power absorbed either in the nanomechanical resonator or the substrate is quantified by obtaining the difference between the transmitted and incident intensity per material stack, and normalizing it with the incident intensity.

## 3. Results and Discussion

### 3.1. Measured Mechanical Spectra

**Figure 2:**
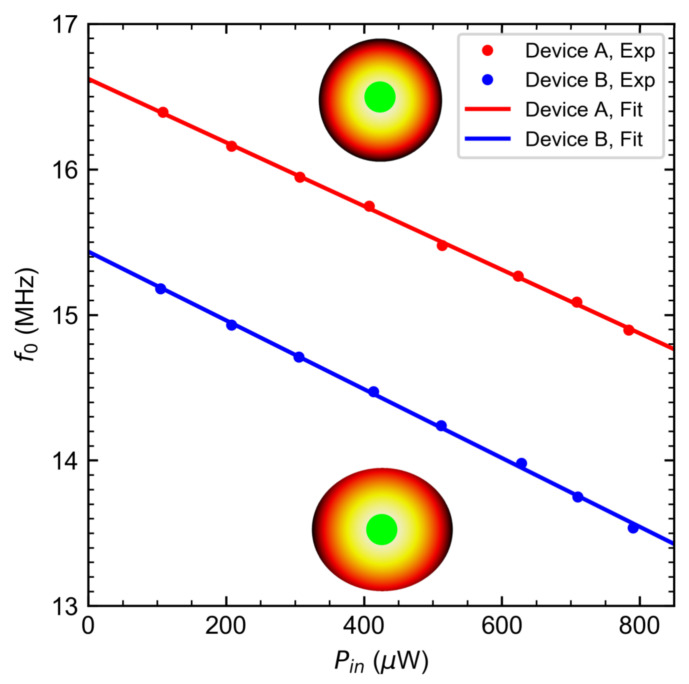
Dependence of the fundamental resonant frequencies of circular (device A) and elliptical (device B) resonators on the incident laser power. Insets show the position of the laser spot (green circle in the center) with a spot diameter of 1.9 μm at which the driven responses of devices A and B are measured. Data points are measured resonant frequencies from the driven responses and solid lines are linear fits. The $f_{0}$ uncertainties from the driven resonator fits are in the order of 1 kHz.

Observation of the photothermal effect on vibrating drums requires changing $P_{in}$ and monitoring the resonance frequency shift in the mechanical spectrum as shown in Figure 1d,e, with amplitude normalized to the incident power. Lightest colored $\tilde{V}/P_{in}$ versus $f_{d}$ responses refer to the driven responses probed with small incident powers, with the darker hue indicating higher probe powers. Figures S1 and S2 of the Supplementary Materials show the raw data of Figure 1d,e, respectively. Variations of $P_{in}$ do not change the shape of the measured response curve, confirming that the drumhead oscillates within the linear regime even at the highest incident laser power [43]. To extract $f_{0}$, the mechanical quality factor $Q_{m}$, and their uncertainties, a linearly-driven damped oscillator model [36,44] is used to fit the measured spectra, with the amplitude $\tilde{V}/P_{in}\propto z$. The number of reported significant figures of $f_{0}$ originates from the standard deviation of the driven damped oscillator model fit and the spacing between driving frequency values. The resulting $f_{0}$ dependences on $P_{in}$ for devices A and B are shown in Figure 2. The $Q_{m}$ of devices A and B for the range of incident powers used are 12.6±0.6 and 23.4±0.3, respectively. A combination of clamping losses, and imperfect, non-uniform boundaries contribute to the low quality factors observed in Figure 1d,e [36].

### 3.2. Concept and Theory

We consider the case of a tensioned circular drumhead where both $D_{p}$ and $\gamma_{0}$ have comparable effect on the resonant frequency of the drumhead. The resonant frequency of both circular and elliptical drumheads, with an effective radius $a_{eff}=\sqrt{ab}$ and thickness *h* can be written as [45]

(5) $$f_{0}\left(P_{in}\right)=\frac{\lambda_{01}}{2\pi }\sqrt{\frac{D_{p}}{\rho ha_{eff}^{4}}\left[\lambda_{01}^{2}+\frac{\left(\gamma_{0}+\gamma_{pth}\right)a_{eff}^{2}}{D_{p}}\right]},$$

where $\lambda_{01}$ is a modal parameter that is determined numerically. At $P_{in}=0$, the drumheads oscillate at their natural resonant frequency (without heating) $f_{0}=f_{0}\left(P_{in}=0\right)$ corresponding to the y-intercept of both plots for circular and elliptical drumheads in Figure 2. Given $f_{0}$ values of 16.62 MHz and 15.43 MHz for device A and B, respectively, and using Equation (5), we determine the initial tension of $\gamma_{0,A}=5.01\;$Nm${}^{-1}$ for device A and $\gamma_{0,B}=5.15\;$Nm${}^{-1}$ for device B, for the given applied DC voltage. As $P_{in}$ ramps up, the downward resonant frequency shift is observed as shown in Figure 2.

Since the frequency shift is linear for small values of $P_{in}$, Equation (5) can be given by its first-order Taylor polynomial

(6) $$f_{0}\left(P_{in}\right)\approx f_{0}+\frac{1}{2}\left[{\left(\frac{\lambda_{01}}{2\pi }\right)}^{2}\frac{1}{\rho ha_{eff}^{2}}\right]\frac{\gamma_{pth}\left(P_{in}\right)}{f_{0}},$$

and the shift can be written as

(7) $$\Delta f_{0}\left(P_{in}\right)=\frac{1}{2}{\left(\frac{\lambda_{01}}{2\pi }\right)}^{2}\frac{\gamma_{pth}\left(P_{in}\right)}{\rho ha_{eff}^{2}f_{0}}.$$

Compressive tension is given by [27,46,47]

(8) $$\gamma_{pth}\left(P_{in}\right)=-\frac{E_{3D}h}{1-\nu }\alpha_{L}\Delta T_{abs}\left(P_{in}\right),$$

where $E_{3D}$ is the Young’s elastic modulus, $\alpha_{L}$ is the thermal expansion coefficient of NbSe_2_ at the bath temperature $T_{0}$, and $\Delta T_{abs}$ is the temperature difference between $T_{0}$ and the average temperature of the drumhead $T_{0}+\Delta T_{abs}$. For linear changes in $T_{0}+\Delta T_{abs}$, the average temperature difference is expressed as

(9) $$\Delta T_{abs}\left(P_{in}\right)=\frac{P_{abs}\left(h,z_{s}\right)}{4\pi h\kappa }\eta ,$$

where κ is the in-plane thermal conductivity of NbSe_2_, and η is the average spot diameter factor. We estimate both χ and $A_{FP}$ through MIA [41,42,48] whereas η is evaluated by assuming absorptive spot heating in the center of the drumheads [49].

Given these inputs, we define the photothermal responsivity Ψ as the frequency shift induced by the absorbed power. By solving Equation (7) using Equations (8) and (9), and defining Ψ as $\Psi =\Delta f_{0}/\Delta P_{in}$, the photothermal responsivity is expressed as

(10) $$\Psi \left(z_{s}\right)=-\frac{1}{8}{\left(\frac{\lambda_{01}}{2\pi }\right)}^{2}\frac{E_{3D}\alpha_{L}}{\left(1-\nu \right)mf_{0}}\frac{\chi A_{FP}\left(h,z_{s}\right)\eta }{\kappa },$$

where $m=\rho \pi a_{eff}^{2}h$ is the total mass of the drumhead. This quantity can be extracted experimentally from the slope of the linear fits acquired from Figure 2. In the tensioned membrane regime, $\Psi \approx \gamma_{0}^{-0.5}$ (see full expression in the Supplementary Materials). This dependence resembles the temperature sensitivity of string-based sensors [50], albeit the thermal tension used to gauge the temperature originated from substrate heating effects. The finding that low $\gamma_{0}$ is favored for temperature sensing due to high temperature sensitivity also applies to photothermal strain sensors based on drumheads. Previous works on interferometric studies of membrane NMRs demonstrate not only imperfections in the amplitude mode shape [51], but also variations of $f_{0}$ with laser beam spot position in the presence of metallic nanoparticles [52]. A localized laser heating study on membranes made from silicon nitride [53] suggests that the spot location should be accounted for when performing power-dependent bolometric tests because applying high $P_{in}$ would induce radial dependence of the measured $f_{0}$. Figure S4 of the Supplementary Materials exhibits that devices A and B show small radial dependences of $f_{0}$ at different incident powers for a 0.2 μm misalignment away from the drum center. The misalignment range is a fraction of the spot diameter, which does not produce significant change in the radial variations of $f_{0}$ at $P_{in}$ of Figure 2. Hence, the power dependence of the frequency shifts within the 0.2 μm radial misalignment range does not significantly deviate from that in Figure 2.

### 3.3. Effect of Static Displacement on the Measured Photothermal Responsivities of NbSe_2_ Drums

**Figure 3:**
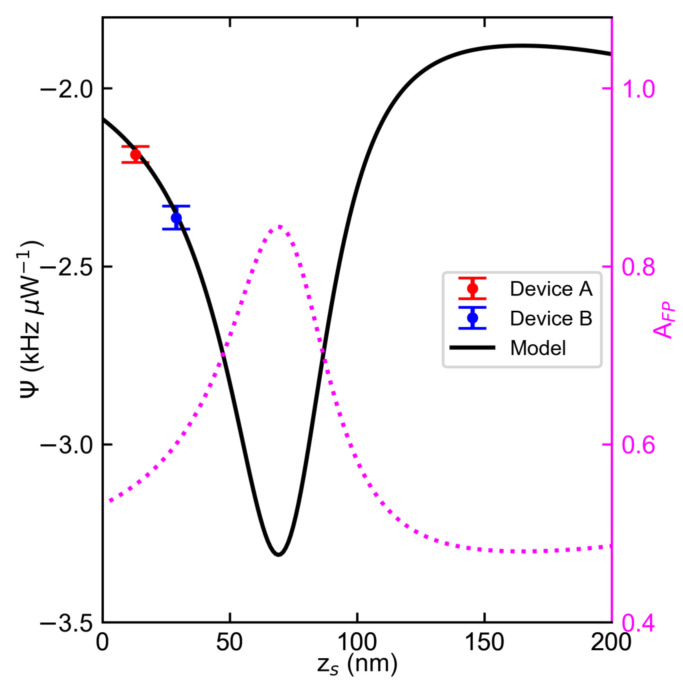
Photothermal responsivity and absorbance dependence of the multilayer NbSe_2_ drumhead resonators on the static displacement. Data points are the slope extracted from the slope in Figure 2. Fitting slope uncertainties in Figure 2 for devices A and B are 35 Hz μW${}^{-1}$ and 22 Hz μW${}^{-1}$, respectively. Solid line refers to the photothermal responsivity model. For comparison, the dependence of the simulated absorbance of the Fabry–Pérot cavity on static displacement, shown by magenta dotted line with the magnitude referenced on the right *Y*-axis spine, is also shown.

Figure 3 shows the dependence of Ψ extracted from Figure 2 with the corresponding $z_{s}$ and $\Psi (z_{s})$ generated with Equation (10) using the resonator specifications and material properties of device A (see Table S1 of the Supplementary Materials). The wavy behavior of $\Psi (z_{s})$ originates from the modulation of the FP absorbance $A_{FP}$ of a 55nm thick bulk NbSe_2_ drumhead as its center moves to a distance $z_{s}$ away from *s*. The described dependence, shown in magenta dotted lines, is simulated using Equation (4). The magnitude of Ψ comes from other material parameters in Equation (4) apart from $A_{FP}$.

The negative Ψ values shown in Figure 3 imply that both devices A and B undergo heat-induced compression. Device A has $\Psi_{A}=-2.19\;$kHz μW${}^{-1}$ for $z_{s}$ = 13nm. Device B has $\Psi_{B}=-2.38\;$kHz μW${}^{-1}$ for $z_{s}=29\;$nm. The ratio of $z_{s}$ between device A and device B in Figure 3 yields 2.23, which is close to the value of 2.16 obtained from the theoretically derived expression $\beta_{ellipse}a_{B}^{4}/\beta_{circle}a_{A}^{4}$, where β is the eccentricity factor from reference [54]. The results imply that the difference in $z_{s}$ of these two drums lies with geometry [8]. Physically, the compressive strain translates to an out-of-plane radial expansion of the drumhead when aided with $z_{s}$. $z_{s}$ is controlled either through electromotive driving of the drumhead [3,55,56] or through slack [8,57,58]. Given the nature of the transfer process using PDMS disks [8,37], $z_{s}$ likely originates from slack.

According to the model prediction in Figure 3, Ψ=−2.10 kHz μW${}^{-1}$ when the resonator is in its equilibrium position ($z_{s}=0$). Displacing the resonator to $z_{s}=69\;$nm, which is a position where a dark FP fringe occurs, results in Ψ≈−3.33 kHz μW${}^{-1}$, which amounts to increases in the measured responsivities of devices A and B by 42% and 52%, respectively. Furthermore, we estimate a tuning range of 1.40  kHz μW${}^{-1}$, which is traced from the $A_{FP}$ difference between the dark and bright fringes.

### 3.4. Simulated Effect of Drumhead Thickness on the Photothermal Responsivity Profile of NbSe_2_ Drums

**Figure 4:**
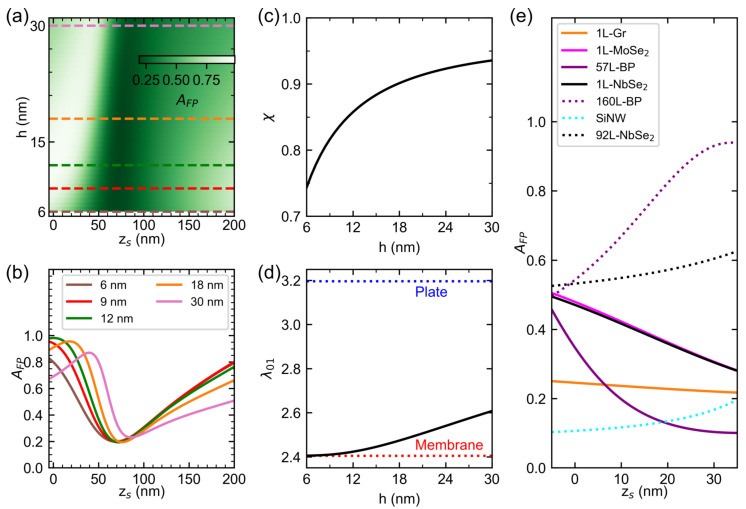
Dependence of the displacement-dependent Fabry–Pérot absorbance profile $A_{FP}\left(z_{s}\right)$ of drum NMRs on the drum thickness and displacement from equilibrium for various resonator materials. (**a**) Intensity color map of the calculated dependence of $A_{FP}\left(z_{s}\right)$ of bulk NbSe_2_ on thickness and displacement from equilibrium. Colored dashed lines correspond to the $A_{FP}\left(z_{s}\right)$ profiles plotted in (**b**). Dependence of the absorbed power fraction χ (**c**) and fundamental mode constant $\lambda_{01}$ (**d**) of bulk NbSe_2_ resonators on thickness. (**e**) Simulated $A_{FP}\left(z_{s}\right)$ profile for different materials. The FP absorbances were simulated for the Fabry–Pérot structure of device A.

**Figure 5:**
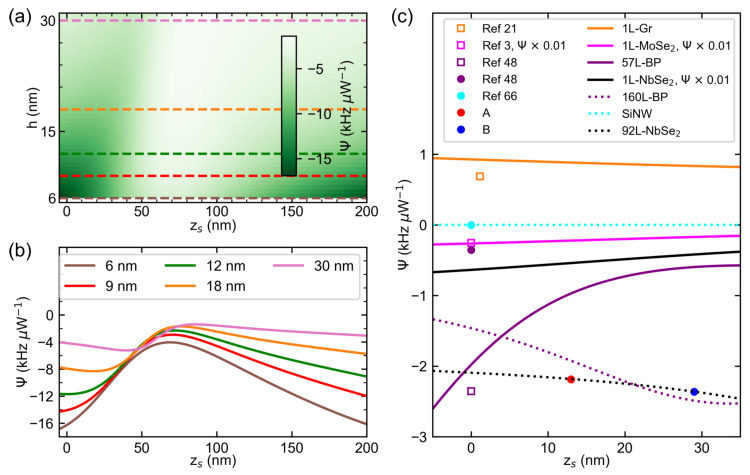
Dependence of the photothermal responsivity profile $\Psi (z_{s})$ of drum NMRs on the thickness and displacement from equilibrium for various resonator materials. (**a**) Intensity plot of the calculated dependence of $\Psi (z_{s})$ of bulk NbSe_2_ drumheads on thickness and displacement from equilibrium. Colored dashed lines correspond to $\Psi (z_{s})$ profiles displayed in (**b**). For comparison, we use the diameter, Fabry–Pérot structure, and mechanical resonant frequency of Device A in simulating the dependences. (**c**) Scatter plot of the photothermal responsivity values obtained in Figure 3, along with photothermal responsivities of other NMRs extracted from the literature. Square symbols refer to devices demonstrating highly tensioned systems such as monolayer (1L) graphene (Gr), monolayer molybdenum diselenide (MoSe_2_), and thin black phosphorus (57L-BP) drumheads. Circular symbols refer to devices dominated by flexural rigidity such as thick black phosphorus (160L-BP) drums, silicon nanowire (SiNW) cantilever beams, and NbSe_2_ drums (A, B). Included also are $\Psi (z_{s})$ of both tension-dominated (solid lines) and flexural rigidity-dominated (dotted lines) devices having the Fabry–Pérot structure of device A.

To further understand the thickness dependence of the photothermal responsivity, we first visualize the effect of *h* on the $A_{FP}\left(z_{s}\right)$ profiles of bulk NbSe_2_ films using Equation (4), as shown in Figure 4a,b. We then simulate the effect of varying *h* on the $\Psi (z_{s})$ profiles of circular bulk NbSe_2_ drumheads using Equation (10), as shown in Figure 5a,b. The simulated $\Psi (z_{s})$ profiles are restricted to the material properties of bulk, clean devices possessing $a_{eff}$, $f_{0}$, FP structure and λ of device A. With these parameters, only $\lambda_{01}$ is varied to decrease at an increasing thickness to maintain $f_{0}$ as shown in Figure 4d. The wavy dependencies of $\Psi (z_{s})$ for multilayer bulk NbSe_2_ originates from the $A_{FP}\left(z_{s}\right)$ plotted in Figure 4a,b. For h=6 nm, we estimate a tuning range of roughly 12.2  kHz μW${}^{-1}$ from $\Psi (z_{s}=0)=-16.30\;$kHz μW${}^{-1}$ to $\Psi (z_{s}=69\;$nm)=−4.05 kHz μW${}^{-1}$. For h=30 nm, we obtain a tuning range of 2.78 kHz μW${}^{-1}$ from $\Psi (z_{s}=0)=-4.16\;$kHz μW${}^{-1}$ to $\Psi (z_{s}=86\;$nm)=−1.39 kHz μW${}^{-1}$. Hence, thinner bulk NbSe_2_ devices have larger tuning range and magnitude as compared to thicker devices. Furthermore, the simulated $\Psi (z_{s})$ profile of monolayer NbSe_2_, shown in Figure 5c as black solid lines, has larger magnitude and tuning ranges than the extrapolated bulk NbSe_2_ $\Psi (z_{s})$ curve (black dotted line).

Intuitively, the change in *h* modifies the properties of both the FP cavity and the nanomechanical resonators, and consequently affects $\Psi (z_{s})$. In the FP domain, *h* modifies both $A_{FP}$ and χ as shown in Figure 4a–c, respectively. In a non-transmissible FP cavity with known refractive indices of the resonators ${\hat{n}}_{res}$[59,60,61,62,63], reflectance and absorbance dominate. For example, the deflection-dependent behavior of the bulk NbSe_2_ $A_{FP}$ transitions from an asymmetric, sinusoidal profile at thin layers to a Fano-peak profile at thicker layers, as shown in Figure 4b. Furthermore, the deflection dependence of monolayer NbSe_2_ $A_{FP}$ maintains an asymmetric, sinusoidal profile due to its large absorption coefficient, which is different compared to bulk NbSe_2_ [36,59]. In device A, the values of $z_{s}$ predicted to have maximum absorbance and magnitude of Ψ would have near-zero modulated reflectance, implying no detectable NMR responses by the FP cavity. Hence the estimates for the tuning range of the drumheads are upper bounds. Nevertheless, the values help confine a range of $z_{s}$ that give detectable driven response and photothermal heating.

At large displacements $z_{s,\mathrm{large}}\geq \lambda /4$, the dependencies of $A_{FP}$ and Ψ on $z_{s,large}$ for NbSe_2_ drums are linear. For 55nm thick drumheads, as shown in Figure 3, both $A_{FP}$ and Ψ are roughly constant at $z_{s,\mathrm{large}}$, indicating the FP cavity produces bright fringes regardless of $z_{s,\mathrm{large}}$. For thinner drumheads described in Figure 4b and Figure 5b, $A_{FP}$ and consequently the magnitude of Ψ increase linearly with $z_{s,\mathrm{large}}$ as the drumheads move to positions where the FP fringe transitions from bright to dark. Furthermore, the slope of the $A_{FP}\left(z_{s,\mathrm{large}}\right)$ profile increases at decreasing *h* and so does the slope of the magnitude of $\Psi (z_{s,\mathrm{large}})$ profile. Beyond $z_{s,\mathrm{large}}\geq 200\;$nm the drumhead would collapse on the electrode when actuated by electromotive force due to pull-in instability [64].

In the nanomechanical resonator domain, the effect of the drum head thickness depends on whether the resonator operates in the low tension or high tension regime (see Supplementary Materials for the full expressions). In the regime where rigidity dominates, $\Psi \propto h^{-2}$. In the tensioned membrane regime in which the modeled multilayer and monolayer NbSe_2_ drums in Figure 5b,c reside, $\Psi \propto h^{-0.5}$. Engineering a constant value of Ψ for any value of $z_{s}$ requires thicker drum plates. For devices with larger tuning ranges of Ψ, thin NbSe_2_ membranes are preferred, though stress-relief structures offer the possibility of thin plate structures [65].

### 3.5. Comparison with Reported Results of Various NMRs from Literature

We note that the $\Psi (z_{s})$ curve of monolayer (1L) NbSe_2_ in Figure 5c represents an upper bound by assuming a clean device ($\rho \approx \rho_{NbSe_{2}}$). These conditions result in a greater magnitude and larger tuning range of $\Psi (z_{s})$ of the monolayer NbSe_2_ than $\Psi (z_{s})$ of other devices such as 165nm thick, clamped-free silicon nanowire (SiNW) resonators [66] (cyan, solid line), graphene (1L-Gr) (orange, solid line), cryogenically-cooled monolayer diselenide (1L-MoSe_2_) (purple, solid line) and multilayer black phosphorus (BP, magenta solid and dotted lines) drums. The shape of these $\Psi (z_{s})$ is reflected in their corresponding $A_{FP}\left(z_{s}\right)$ dependencies as shown in Figure 4e, which uses the substrate and λ of device A. For the properties of the above-listed materials that define the magnitude of $\Psi (z_{s})$, see Tables S1 and S2 of the Supplementary Materials. Note that the $\Psi (z_{s})$ profiles for BP resonators assume an average, isotropic $E_{3D}$ and κ even though these resonators have reported anisotropic properties [48]. The indicated materials have positive $\alpha_{L}$ which implies that the devices made of these materials experience compression upon the illumination by the laser beam. Only the $\Psi (z_{s})$ curve of graphene experiences tension upon spot heating due to graphene’s negative $\alpha_{L}$ at ambient temperature. Furthermore, the SiNW device falls under the optically-thick, bending regime, which illustrates the near-zero magnitude and tunability of Ψ with $z_{s}$.

We also see from Figure 5c that the measured Ψ of bulk NbSe_2_ devices is comparable to that of 57L-BP device (magenta square) and significantly better than both bulk SiNW (cyan circle) and 160L-BP (magenta circle) devices. However, Ψ’s of these devices are ten times smaller than the Ψ values of a 1L-MoSe${}_{2}$ drumhead (pink square), which possesses a significantly smaller mass. We note that the values of $z_{s}$ extracted from the literature are for the devices in their equilibrium position except for graphene, which has 1 nm deflection to resolve its motion [21].

The effect of the substrate and probing wavelength on the photothermal responsivity of the drumheads varies with the resonator material as its optical, thermal, and mechanical properties modify their $\Psi (z_{s})$ profiles. For example, the highly-reflective substrate of device A shows improved $\Psi (z_{s}=0)$ for the 160L-BP, 1L-Gr and 1L-MoSe${}_{2}$ devices, comparable $\Psi (z_{s}=0)$ to the SiNW device and decreased $\Psi (z_{s}=0)$ for the 57L-BP drumhead as compared to their extracted values in the literature [3,21,48,66] (colored symbols), as shown in Figure 5c. The devices cited in the literature make use of a combination of gap heights, probe wavelength, and substrate for near-optimal FP-based detection [67] and ease in device fabrication; their photothermal responses are secondary. Nevertheless, our model demonstrates the variation of the tuning range of $\Psi (z_{s})$ of thin van der Waals materials using our FP structure.

The values of $\Psi (z_{s})$ reported in this work, with the exception of clean 1L-NbSe_2_, are fractions of Ψ reported for metamaterial string [31] and graphene trampoline NMR bolometers [27] at infrared and visible wavelengths, respectively. Apart from replacing NbSe_2_ with thermally insulating vdW materials, a strategy to improve $\Psi (z_{s}=0)$ of NbSe${}_{2}$ involves decreasing the thermal conductivity of the drumheads with high-aspect-ratio tethers that resemble a trampoline geometry. The trampoline structure is reported to increase $\Psi (z_{s}=0)$ of graphene, known for its very high thermal conductivity, to $2.4\times 10^{3}\;$kHz μW${}^{-1}$ [27]. The structure is tricky to implement on NbSe_2_ since the focused ion beam etching step introduces defects that alter the flake properties [68,69]. On another note, broadening the tuning range would entail engineering a partially-transparent substrate at a probe wavelength to distinguish the resonator positions of maximum absorbance and zero modulated reflectance [20]. Finally, the proposed model guides the design and fabrication of FP-based, ultrathin nanomechanical bolometers made from NbSe_2_ and other vdW materials, whose photothermal responsivity is tunable with static displacement. Future directions include experimental demonstrations of the dependence of vdW NMR photothermal responsivity on the drumhead thickness and initial tension.

## 4. Conclusions

In summary, we have designed and characterized NbSe_2_ drumheads with controllable photothermal heating using NMR static displacements in FP cavities. Our simulations show that the magnitude and tuning range of the photothermal response of drumheads increase at decreasing flake thickness. Our analysis shows that a monolayer NbSe_2_ drumhead NMR has promising photothermal responsivities at room temperature, while our work focuses on NbSe_2_ devices, the design framework applies to a family of vdW materials and conventional resonator materials that absorb light.

## Data Availability

Data and additional details are contained within the article and in the Supplementary Materials.

## Supplementary Materials

The following supporting information can be downloaded from: https://www.mdpi.com/article/10.3390/nano12152675/s1: Table S1: Parameters of vdW drumhead NMRs used in the calculation of the FP absorbances in Figure 3 and Figure 4a,b,e, and photothermal responsivities in Figure 3 and Figure 5a–c; Table S2: Parameters used in the calculation of FP absorbance and photothermal responsivities of silicon nanowire resonators in Figure 4e and Figure 5c, respectively; Figure S1: Raw amplitude and phase response of device A; Figure S2: Raw amplitude and phase response of device B; Figure S3: Simulated temperature variation of device A; Figure S4: Dependences of the fundamental mode frequency on the laser spot position of devices A and B within twice of the misalignment range; Supplementary Material Section S1: Derivation of Photothermal Responsivities; Supplementary Material Section S2: Observed Dependencies of $f_{0}$ on the Laser Spot Position; Supplementary Material Section S3: Effect of Radiation Pressure. References [70,71,72,73,74,75,76,77,78,79,80,81,82,83,84,85,86,87,88,89,90,91,92,93,94,95,96,97,98] are cited in the Supplementary Materials.
