# Supplementary material for: Photothermal Responsivity of van der Waals Material-Based Nanomechanical Resonators
Source: Nanomaterials (Basel). 2022 Aug 4;12(15):2675. doi: 10.3390/nano12152675 (PMC9370576; doi:10.3390/nano12152675)
Supplement: Supplementary file 1 [file nanomaterials-12-02675-s001.zip › main20220804_SI.pdf]

Supplementary Materials: Photothermal Responsivity of van der Waals Material-Based Nanomechanical Resonators

Myrron Albert Callera Aguila [1,2,3,*], Joshoua Condicion Esmenda [1,2,3], Jyh-Yang Wang [3], Yen-Chun Chen [3], Teik-Hui Lee [3], Chi-Yuan Yang [3], Kung-Hsuan Lin [3], Kuei-Shu Chang-Liao [1], Sergey Kafanov [4], Yuri A. Pashkin [4] and Chii-Dong Chen [3,*]

Table S1. Parameters of vdW drumhead NMRs used in the calculation of the FP absorbances in Figure 3 and Figures 4(a,b,e), and photothermal responsivities in Figure 3 and Figures 5(a-c) of the main text

Materials	$NbSe_2$	Graphene [1]	$MoSe_2$ [2]	Black Phosphorus[b] [3]
a_{eff} μm	3.5	5.5	1.25	4.5 (≈160L), 4 (≈57L)
h_{1L} (nm)	0.6 [4]	0.335 [1]	0.65 [5]	0.53 [6]
T (K)	293	293	50	300
E_Y (GPa)	100 [7]	630[a] [1]	160 [8]	22 (AC) [3], 72 (ZZ) [3]<br>47 (Mean)
ρ (kg m^{-3})	6467 [9]	2200 [1]	6990 [10]<br>$\times$ 7.98[a]	2690 [11]
ν	0.24 [12]	0.16 [1]	0.18 [13]	0.73 (AC) [14], 0.17 (ZZ) [14]<br>0.45 (Mean)
$\alpha_L(T)$ (10^{-6} K^{-1})	4.1 [15]	−7 [16]	2.1 [8]	5.5 [3]
κ (W m^{-1} K^{-1})	14.5 [17,18]	5000 [19]	28 [2]	13 (≈160L), 9 (≈57L) (AC) [3]<br>49 (≈160L), 37 (≈57L) (ZZ) [3]<br>31 (≈160L), 23 (≈57L) (Mean)
$\hat{n}_{res}(\lambda$=532 nm)	6.48 − 1.69j (1L) [20]<br>3.07 − 1.00j (Bulk) [20]	2.68 − 1.22j [21]	5.21 − 0.93j [22,23]	2.65 − 0.60j (AC) [24]<br>2.69 − 0.25j (ZZ) [24]<br>2.67 − 0.43j (Mean)
χ	0.491 (1L)<br>0.958 (92L)	0.140	0.515	0.848 (≈ 57L)<br>0.908 (≈ 160L)
m_{tot} (10^{-18}kg)	149 (1L)<br>1.37×10^4 (92L)	73.2	177	4.06×10^3 (≈57L)<br>1.45×10^4 (≈160L)
$f_0(P_{in}=0)$ (MHz)	16.62	4.76	33.03	8.397 (≈57L)<br>9.313 (≈160L)
Ψ_{exp} (kHz μW^{-1})	see main text	0.68[a] [1]	−25.47[a] [2]	−2.35 (≈57L) [3]<br>−0.36 (≈160L) [3]

[a] Inferred from Supplementary Material of cited reference
[b] Estimation of Ψ assumes an isotropic material even though the device has anisotropic properties in the armchair (AC) and zigzag (ZZ) orientations. Average values of E_Y, ν, κ and $\hat{n}$ were taken.

1. Derivation of Photothermal Responsivities

1.1. Clamped Circular Drumheads

To gain insight into how laser-induced photothermal tension affects the fundamental mode frequency of a $NbSe_2$ drumhead device, we model the drumhead as a tensioned plate whose resonant frequency accounts for both flexural rigidity $D_p = E_{3D}h^3/12(1-\nu^2)$ and tension γ. Here, E_{3D} is the Young's elastic modulus, ν is the Poisson's ratio, and h is the thickness. The frequency of the fundamental mode can then be written as [30]

$$f_0(P_{in}) = \sqrt{\left(\frac{\lambda_{01}}{2\pi}\right)^2 \left[\frac{\lambda_{01}^2 D_p}{\rho h a_{eff}^4} + \frac{\gamma_{tot}}{\rho h a_{eff}^2}\right]}, \tag{S1}$$

where a_{eff} is the effective radius of the drumhead, $\rho = 6467$ kg m^{-3} is the mass density of $NbSe_2$, λ_{01} is a modal parameter of the fundamental mode that describes not only both

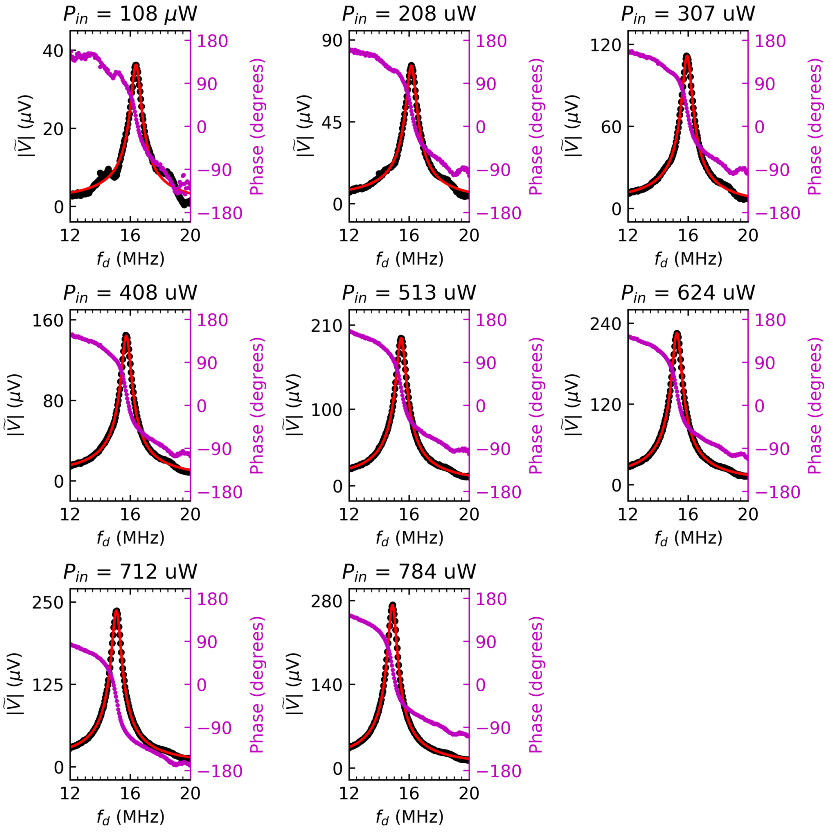

Figure S1. Raw amplitude (black dots) and phase (magenta dots) response of device A. Red solid lines are fits using the linearly driven damped resonator model.

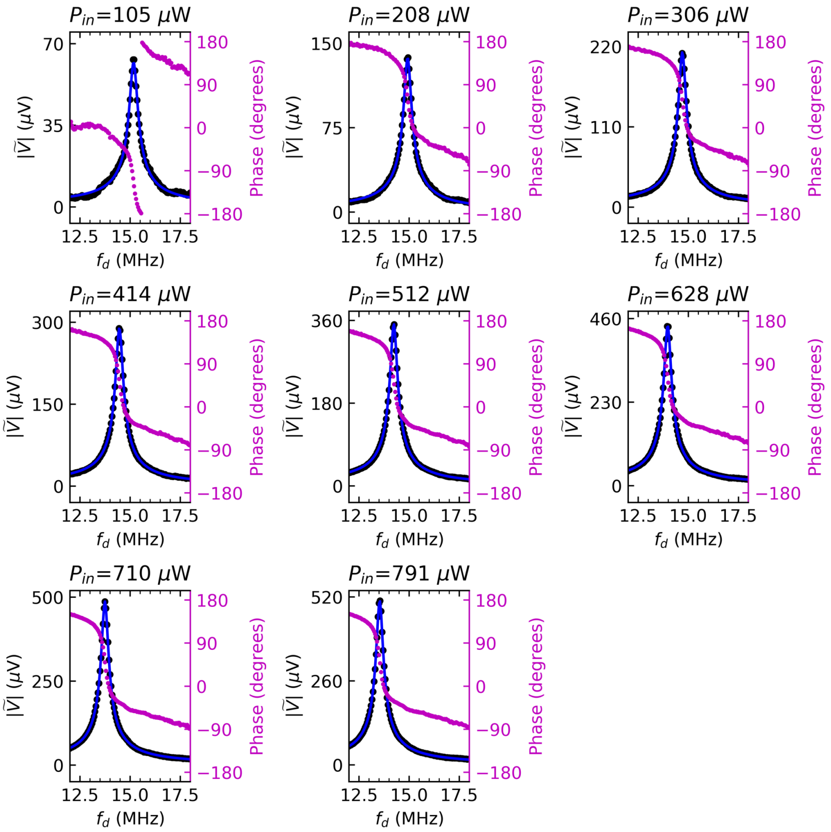

Figure S2. Raw amplitude (black dots) and phase (magenta dots) response of device B. Blue solid lines are fits using the linearly driven damped resonator model.

Table S2. Parameters used in the calculation of FP absorbance and photothermal responsivities of silicon nanowire resonators [25] in Figure 4(e) and Figure 5(c) of the main text

Parameters	Values	Notation and Units
Length	12.7	l (μm)
Cross-section Diameter / Thickness	165	$d = h$ (nm)
Base Temperature	293	T (K)
Elastic Modulus	170 [26]	E_{3D} (GPa)
Poisson's Ratio	0.28 [26]	ν
Density	2329 [27]	ρ (kg m^{-3})
Thermal Expansion Coefficient	2.6 [28]	$\alpha_L(T)$ (10^{-6} K^{-1})
Thermal Conductivity	131 [27]	κ (W m^{-1} K^{-1})
Refractive Index at $\lambda = 532$ nm	4.15 − 0.04j [29]	$\hat{n}_{res}(\lambda = 532\,\text{nm})$
Absorbed Power Fraction	0.709	χ
Total Mass	632	m_{tot} (10^{-18}kg)
Fundamental Frequency	1.7442	$f_1(P_{in} = 0)$ (MHz)
Experimental Phothermal Responsivity	0.17×10^{-3}	Ψ_{exp} (kHz μW^{-1})

tension-dominant and tension-free limits, but also their cross-over regimes [31,32]. The total tension can be presented as a sum $\gamma_{tot} = \gamma_0 + \gamma_{pth}(P_{in})$, where γ_0 is the tension exerted on the drumhead by the clamps during the fabrication process and $\gamma_{pth}(P_{in})$ is the radial tension induced by the incident laser power P_{in}. For highly optically absorptive drumheads, radiation pressure effects are negligible. We then re-write Eq. (S1) as

$$f_0(P_{in}) = \sqrt{\left(\frac{\lambda_{01}}{2\pi}\right)^2 \left[\frac{\lambda_{01}^2 D_p}{\rho h a_{eff}^4} + \frac{\gamma_0}{\rho h a_{eff}^2}\right] + \left(\frac{\lambda_{01}}{2\pi}\right)^2 \frac{\gamma_{pth}(P_{in})}{\rho h a_{eff}^2}}. \tag{S2}$$

Because the linear frequency dependence on P_{in} is observed, Eq. (S2) can be approximated as a first order Taylor series expansion in the form $f_0(P_{in}) \approx \sqrt{f_0^2} + (1/2)B\gamma_{pth}(P_{in})/\sqrt{f_0^2}$, where $f_0 = f_0(P_{in} = 0)$ and $B = (\lambda_{01}/2\pi)^2[\rho h a_{eff}^2]^{-1}$. Extrapolating the input powers to $P_{in} = 0$ allows the determination of γ_0 via numerical means [30]. For simplicity, both minor and major diameters are represented by $a_{eff} = \sqrt{ab}$. The expected frequency shift Δf_0 is then given by

$$\Delta f_0(P_{in}) \approx \left[f_0 + \frac{1}{2}\left(\frac{\lambda_{01}}{2\pi}\right)^2 \frac{\gamma_{pth}(P_{in})}{\rho h a_{eff}^2}\right] - f_0. \tag{S3}$$

Simplifying the expression leads to

$$\Delta f_0(P_{in}) \approx \frac{1}{2}\left(\frac{\lambda_{01}}{2\pi}\right)^2 \frac{\gamma_{pth}(P_{in})}{\rho h a_{eff}^2 f_0}. \tag{S4}$$

It is assumed in this work that heat convection and radiation do not contribute to heat transport in resonators with metallic thin film absorbers [33], leaving heat transfer due to thermal conductivity as the major mechanism for creating thermal stress. The laser spot in the drum center creates a localized heat source with heat flowing radially to the clamps. For materials with a positive thermal expansion coefficient α_L, the differential temperature between the drumhead and the drum clamps enables thermal expansion of the drum when unrestrained. The clamps, being fixed supports, exert a compressive stress on the drum to maintain the drum diameter. As a result, the thermal expansion introduces compressive strain [3,34]. Thermally induced tension reduces the built-in tension, as observed in doubly-clamped beam [35], plate [3], and membrane [28,33] resonators with nanoscale dimensions. As the frequency shift is linear, the heat-induced strain is also linear, and so is the average

differential temperature ΔT_{abs} between the hot spot and the drum clamps. The thermally induced tension is then expressed as [36,37]

$$\gamma_{pth}(P_{in}) = -\frac{E_{3D}h}{1-\nu}\alpha_L \Delta T_{abs}(P_{in}), \tag{S5}$$

α_L is assumed to be constant in the range of temperatures induced by the absorbed laser power.

To obtain the differential temperature profile of the drumhead, we first assume that the incident laser spot does not significantly change the thermal conductivity κ in the range of P_{in} applied in the experiment. In the first-principles study by Ferreiro [18], the phonon mean free path of 1L $NbSe_2$ was estimated to be 2.5 μm at ambient temperature, suggesting that heat transport in our drumheads is diffusive. Multilayered vdW materials show low out-of-plane thermal conductivity [38,39], enabling us to ignore heat conduction between vdW layers. In addition, heat conduction between the flake and the spacer is negligible due to the low thermal conductivity of the polymeric spacer [40]. These boundary conditions enable the radial temperature profile of the drumhead to be solved using a 1-D radial heat diffusion equation of a thin circular disk [41]

$$\frac{1}{r}\frac{d}{dr}\left(r\frac{d\Delta T}{dr}\right) + \frac{P_{abs}(h, z_s)}{\pi r_0^2 h\kappa} \times H(r - r_0) = 0, \tag{S6}$$

where κ is the in-plane thermal conductivity, $H(r - r_0)$ is the Heaviside step function that represents the illumination of a laser beam of spot diameter $2r_0 = 1.9\,\mu m$ such that $a_{eff} > r_0 > 0$, and P_{abs} is the power absorbed by the resonator from the laser beam.

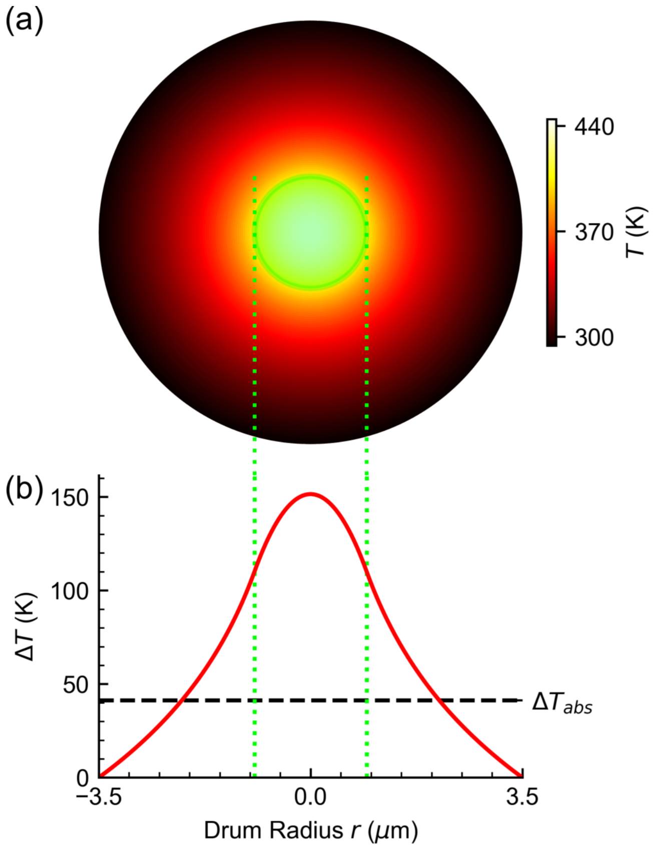

Figure S3. (a) Simulated temperature variation of the drumhead, with its center illuminated by a laser beam whose absorbed power is $P_{abs} = 417\,\mu$W. The green spot refers to the incident laser spot that acts as a localized heat source. The spot diameter is 1.9μm. (b) Simulated differential temperature profile across the drumhead diameter. ΔT_{abs} is the average differential temperature of the drumhead.

A linear change in the photothermal strain translates to a small differential temperature and an averaged in-plane thermal conductivity between the range of temperatures induced by P_{abs}. Given that $\Delta T = \Delta T(r)$ and $\Delta T(r = a_{eff}) = 0$, the solution of Eq. (S6) for the radial differential temperature profile has the form [42,43]

$$\Delta T(r) = \begin{cases} M\left[\left(1 - r^2/r_0^2\right) - 2\ln\left(r_0/a_{eff}\right)\right] & \text{for } 0 \leq \mathrm{r} \leq \mathrm{r}_0, \\ -2M\ln\left(r/a_{eff}\right) & \text{for } \mathrm{r}_0 \leq \mathrm{r} \leq \mathrm{a}_{\mathrm{eff}}, \end{cases} \tag{S7}$$

where $M = P_{abs}(h, z_s)/(4\pi\kappa h)$. Temperature variation $T(r)$ and a cross-section profile of Eq. (S7) are plotted in Figure S3 along with the average temperature difference ΔT_{abs} induced by the laser beam. ΔT_{abs} is the average of the differential temperature across the circular disk area defined as [41]

$$\Delta T_{abs} = \frac{2\pi\int_0^{a_{eff}} r\Delta T(r)dr}{2\pi\int_0^{a_{eff}} rdr} = \frac{\left[\int_0^{r_0} r\Delta T(r)dr + \int_{r_0}^{a_{eff}} r\Delta T(r)dr\right]}{\left(a_{eff}^2/2\right)}. \tag{S8}$$

Evaluating Eq. (S8) with Eq. (S7), ΔT_{abs} is then simplified as [41]

$$\Delta T_{abs}(P_{in}) = T_{avg} - T_0 = \frac{P_{abs}(h, z_s)}{4\pi\kappa h}\eta. \tag{S9}$$

where η is defined as the average spot diameter factor. Both devices A and B show $\eta \approx 1$, which implies that the spot diameter is small enough to heat the entire membrane with the absorbed power transferred from the FP cavity. Eq. (S9) enables us to express the radial tension in Eq. (S5) as

$$\gamma_{pth}(P_{in}) = -\frac{E_{3D}\alpha_L}{1-\nu}\left(\frac{P_{abs}(h, z_s)}{4\pi\kappa}\eta\right), \tag{S10}$$

The photothermal-mediated frequency shift in Eq. (S4) can be written as

$$\Delta f_0(P_{in}) \approx -\frac{1}{8}\left(\frac{\lambda_{01}}{2\pi}\right)^2 \frac{E_{3D}\alpha_L}{(1-\nu)mf_0}\left(\frac{P_{abs}(h, z_s)\eta}{\kappa}\right), \tag{S11}$$

where $m = \rho\pi a_{eff}^2 h$ is the total mass of the device. Finally, we define the photothermal responsivity Ψ of a multilayered tensioned plate as

$$\Psi(z_s) = \frac{\Delta f_0}{\Delta P_{in}} = -\frac{1}{8}\left(\frac{\lambda_{01}}{2\pi}\right)^2 \frac{E_{3D}\alpha_L}{(1-\nu)mf_0}\frac{\chi A_{FP}(h, z_s)\eta}{\kappa}. \tag{S12}$$

For the bulk and monolayer $NbSe_2$ drumheads, the thermal conductivity that fits the measured frequency dependence shown in Figure 2 of the main text is $\kappa = 14.5\,\mathrm{W\,m^{-1}\,K^{-1}}$, which is close to the values reported elsewhere [17,18].

For an intuitive understanding of the dependence of Ψ on the resonator parameters a_{eff}, h and γ_0, we evaluate Ψ both in the bending limit ($D_p \gg \gamma_0 a_{eff}^2$) and in the high tension limit ($D_p \ll \gamma_0 a_{eff}^2$). In the bending limit, the photothermal responsivity of a circular nanomechanical plate is

$$\Psi_{plt}(z_s) = -\frac{\sqrt{3}}{8\pi^2}\sqrt{\frac{E_{3D}(1+\nu)}{\rho(1-\nu)}}\frac{1}{h^2}\frac{\alpha_L\chi A_{FP}(h, z_s)\eta}{\kappa}. \tag{S13}$$

Eq. (S13) shows that Ψ_{plt} scales as $1/h^2$ and does not depend on a_{eff}. The measured drumheads and the 160L-BP drumhead follows Eq. (S13).

In the high tension limit, the photothermal responsivity of a circular membrane is

$$\Psi_{mem}(z_s) = -\frac{\lambda_{01,mem}}{16\pi^2}\frac{E_{3D}}{(1-\nu)\sqrt{\rho\gamma_0}}\frac{1}{\sqrt{h}a_{eff}}\frac{\alpha_L\chi A_{FP}(h,z_s)\eta}{\kappa}, \quad (S14)$$

where $\lambda_{01,mem} = 2.4$ is the fundamental mode constant of a circular membrane. Eq. (S14) shows that Ψ_{mem} scales as $1/(\sqrt{h}a_{eff})$. Thinner $NbSe_2$ (shown in Figure 5 in the main text), 57L-BP, 1L-Gr and 1L-$MoSe_2$ drumheads follow Eq. (S14). When Eq. (S14) is divided by the resonant frequency of a tensioned membrane, the resulting expression is independent of a_{eff}, similar to the expression reported elsewhere [33,42].

1.2. Clamped-Free Cantilever Beams

For a clamped-free cantilever beam with length l and cross-sectional diameter $d = h$ such that $d \ll 2r_0 < l$, we define a participation factor $\beta = 2\pi(d/2)\sqrt{(2r_0)^2 - d^2}/\pi r_0^2 \approx 2d/r_0$, that serves as a prefactor to the absorbed power P_{abs}. Since the probe laser is focused on the free end of the cantilever, we assume linear differential temperature profile across the beam [44]. The resonant frequency of a cantilever beam with added curvature (or under surface stress) defined by Δl_0 is described as [45,46]

$$f_1(P_{in}) = \frac{\alpha_1^2}{4\pi\sqrt{3}}\sqrt{\frac{E_{3D}}{\rho}}\frac{d}{(l+\Delta l_0+\Delta l(P_{in}))^2} \approx 0.162\sqrt{\frac{E_{3D}}{\rho}}\frac{d}{(l+\Delta l_0+\Delta l(P_{in}))^2}. \quad (S15)$$

Expanding Eq. (S15) to its first order Taylor-series expansion term defines the change of the resonant frequency for a cantilever beam due to laser induced heating as

$$\Delta f_1(P_{in}) = \frac{f_1^{3/2}(\Delta l_0)}{f_1^{1/2}(\Delta l_0 = 0)}(-2\alpha_L\Delta T_{cant}(P_{in})), \quad (S16)$$

where ΔT_{cant} is the average cantilever temperature. Before determining ΔT_{cant}, the lateral temperature profile of the cantilever beam is solved using a 1-D heat diffusion equation of a clamped-free cylindrical rod

$$\frac{d^2\Delta T_c}{dx^2} + \frac{P_{abs}(d,z_s)}{2\pi\kappa d^2 r_0} \times \mathrm{H}(x-[l-r_0]) = 0, \quad (S17)$$

where $H(x-[l-r_0])$ is the Heaviside step function that represents the illumination of a laser beam on the free end of the cantilever ($x = l - r_0$). Given that $\Delta T_c = \Delta T(x)$, $\Delta T_c(x=0) = 0$, $\Delta T_c(x = l-2r_0) = \Delta T_c(x = l)$ and $d(\Delta T_c(x = l - r_0))/dx = 0$, the solution for the differential temperature profile of the laser heated cantilever has the form [47]

$$\Delta T(x) = \begin{cases} M_{cant}(2x/d) & \text{for } 0 \le x \le l - 2r_0, \\ (M_{cant}/d)\left(r_0\left[1-((x-[l-r_0])/r_0)^2\right] + 2[l-2r_0]\right) & \text{for } l-2r_0 \le x \le l, \end{cases} \quad (S18)$$

where $M_{cant} = P_{abs}(d,z_s)/(4\pi\kappa d)$. ΔT_{cant} is the average temperature across the cantilever defined as

$$\Delta T_{cant} = T_{avg} - T_0 = \frac{\int_0^l \Delta T(x)dx}{\int_0^l dx} = \frac{\left[\int_0^{l-2r_0}\Delta T(x)dr + \int_{l-2r_0}^{l}\Delta T(x)dx\right]}{l} = \frac{P_{abs}(d,z_s)}{4\pi\kappa d}\eta_c, \quad (S19)$$

where $\eta_c = 76.4$. Combining Eq. (S16) and Eq. (S19) and dividing by the incident power P_{in}, we define the photothermal sensitivity of the cantilever as

$$\Psi_{cant}(z_s) = -\frac{f_l^{3/2}(\Delta l_0)}{f_1^{1/2}(\Delta l_0 = 0)}\left(\frac{\alpha_L \beta \chi A_{FP}(d, z_s)}{2\pi\kappa d}\right)\eta_c, \quad \text{(S20)}$$

where $\beta = 0.355$. This expression can be evaluated by using the resonator dimensions to define $f_1^{1/2}(\Delta l_0 = 0)$ and using the measured out-of-plane resonant frequency of the silicon nanowire [25] to define $f_1^{3/2}(\Delta l_0)$. Evaluation of Eq. (S20) for a range of z_s represents the cyan dotted line in Figure 5(c) of the main text.

2. Observed Dependence of f_0 on the Laser Spot Position

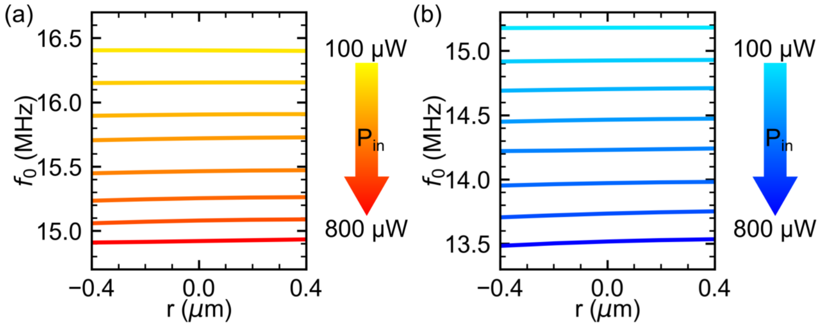

Figure S4. Dependence of the fundamental mode frequency on the laser spot shift from the center of devices A (a) and B (b). The change of colors from light to dark corresponds to the power increase from 100 μW to 800 μW as noted in Figure 2 of the main text.

Previous works on spatial mode mapping of membrane NMRs show asymmetry in the fundamental mode shapes due to polymeric adsorbates [48] and variations of f_0 across the resonator dimensions due to the presence of metallic nanoparticles [49]. Recent work on membranes made from silicon nitride, a non-absorbing material, demonstrates that the laser beam spot position affects the radial dependence of f_0 at high incident laser power, having a frequency shift of 0.7 kHz at the center of the membrane [28]. The work suggests that the observed frequency shifts due to laser power are dependent on the laser beam spot location, and both the laser beam spot diameter and alignment are accounted for when performing power-dependent bolometric studies on NMRs. Figure S4(a,b) shows the dependence of f_0 of devices A and B on the radial position of the laser spot, which moves from the $NbSe_2$ drumhead center. Within twice the observed misalignment range of roughly 0.2 μm from the center, the radial dependence of f_0 for devices A and B are small even at the highest incident power. Figure S4 suggests that the observed frequency shifts in Figure 2 of the main text are not significantly affected by laser beam spot misalignment.

3. Effect of Radiation Pressure

The radiation pressure of an incident laser beam can be written as [50]

$$\sigma_{rad,in} = \frac{I_{rad}}{c} \quad \text{(S21)}$$

where I_{rad} is the incident irradiance (W m^{-2}) and c is the speed of light. Given an incident power $P_{in} \approx 800\,\mu$W and the spot diameter $2r_0 = 1.9\,\mu$m, the stress due to the incident radiation pressure is about 0.99 Pa, which is equivalent to a tension [51] of $\gamma_{rad,in} = \sigma_{rad,in} h = 55\,\text{nN}\,m^{-1}$ for 55 nm thick drumheads.

The maximum radiation pressure exerted by a Fabry–Pérot interferometer onto the vibrating drumhead depends on the reflectance of the $NbSe_2$ flake and the bottom electrode. The maximum force gradient of radiation pressure is written as [1,52]

$$\nabla F_{rad,max} = \gamma_{FP,max} \approx \frac{2P_{in}}{c\lambda}\left(2\sqrt{R_{res}}\left[\frac{4R}{(1-R)^2}\right]\right), \tag{S22}$$

where $\lambda = 532\,\text{nm}$ is the laser wavelength, and R is the reflectance described as

$$R = \sqrt{R_{res}R_{sub}}, \tag{S23}$$

where R_{res} is the reflectance of the $NbSe_2$ flake and R_{sub} is the reflectance of the bottom mirror. As the $NbSe_2$ drums have non-negligible thickness, and the bottom mirror is a stack of reflective and transparent materials, their reflectances are calculated via MIA [53,54] to obtain $R_{res} = 0.408$ and $R_{sub} = 0.761$. From we obtain Eq. (S22), $\gamma_{FP,max} = 0.14$ mN m^{-1}, which is roughly 2.6 kPa when converted to stress. These estimates, which are much smaller than the initial tension of both drums, imply that radiation pressure does not affect noticeably the reported results.

References

1. Barton, R.A.; Storch, I.R.; Adiga, V.P.; Sakakibara, R.; Cipriany, B.R.; Ilic, B.; Wang, S.P.; Ong, P.; McEuen, P.L.; Parpia, J.M.; et al. Photothermal Self-Oscillation and Laser Cooling of Graphene Optomechanical Systems. *Nano Lett.* **2012**, *12*, 4681–4686. doi:10.1021/nl302036x.
2. Morell, N.; Tepsic, S.; Reserbat-Plantey, A.; Cepellotti, A.; Manca, M.; Epstein, I.; Isacsson, A.; Marie, X.; Mauri, F.; Bachtold, A. Optomechanical Measurement of Thermal Transport in Two-Dimensional $MoSe_2$ Lattices. *Nano Lett.* **2019**, *19*, 3143–3150. doi:10.1021/acs.nanolett.9b00560.
3. Islam, A.; van den Akker, A.; Feng, P.X. Anisotropic Thermal Conductivity of Suspended Black Phosphorus Probed by Opto-Thermomechanical Resonance Spectromicroscopy. *Nano Lett.* **2018**, *18*, 7683 – 7691. doi:10.1021/acs.nanolett.8b03333.
4. Castellanos-Gomez, A.; Agraït, N.; Rubio-Bollinger, G. Optical Identification of Atomically Thin Dichalcogenide Crystals. *Appl. Phys. Lett.* **2010**, *96*, 213116. doi:10.1063/1.3442495.
5. Shaw, J.C.; Zhou, H.; Chen, Y.; Weiss, N.O.; Liu, Y.; Huang, Y.; Duan, X. Chemical Vapor Deposition Growth of Monolayer $MoSe_2$ Nanosheets. *Nano Res.* **2015**, *7*, 511–517. doi:10.1007/s12274-014-0417-z.
6. Xia, F.; Wang, H.; Jia, Y. Rediscovering Black Phosphorus as an Anisotropic Layered Material for Optoelectronics and Electronics. *Nat. Commun.* **2014**, *5*, 4458. doi:10.1038/ncomms5458.
7. Sengupta, S.; Solanki, H.S.; Singh, V.; Dhara, S.; Deshmukh, M.M. Electromechanical Resonators as Probes of the Charge Density Wave Transition at the Nanoscale in $NbSe_2$. *Phys. Rev. B.* **2010**, *82*, 155432. doi:10.1103/PhysRevB.82.155432.
8. Çakır, D.; Peeters, F.M.; Sevik, C. Mechanical and Thermal Properties of h-MX_2 (M = Cr, Mo, W; X = O, S, Se, Te) Monolayers: A Comparative Study. *Appl. Phys. Lett.* **2014**, *104*, 203110. doi:10.1063/1.4879543.
9. Meerschaut, A.; Deudon, C. Crystal Structure Studies of the $3R-NbSe_{1.09}S_2$ and the $2H-NbSe_2$ Compounds: Correlation between Nonstoichiometry and Stacking Type (= Polytypism). *Mater. Res. Bull.* **2001**, *36*, 1721–1727. doi:10.1016/S0025-5408(01)00646-8.
10. $MoSe_2$ Crystal Structure: Datasheet from "PAULING FILE Multinaries Edition – 2012" in Springer Materials. Copyright 2016 Springer-Verlag Berlin Heidelberg & Material Phases Data System (MPDS), Switzerland & National Institute for Materials Science (NIMS), Japan (https://materials.springer.com/isp/crystallographic/docs/sd_0454362).
11. Liu, H.; Du, Y.; Deng, Y.; Ye, P.D. Semiconducting Black Phosphorus: Synthesis, Transport Properties and Electronic Applications. *Chem. Soc. Rev.* **2015**, *44*, 2732–2743. doi:10.1039/c4cs00257a.
12. Persson, K. Materials Data on $NbSe_2$ (SG:194) by Materials Project, 2016. doi:10.17188/1197331.
13. Persson, K. Materials Data on $MoSe_2$ (SG:194) by Materials Project, 2014. doi:10.17188/1191826.
14. Wei, Q.; Peng, X. Superior Mechanical Flexibility of Phosphorene and Few-Layer Black Phosphorus. *Appl. Phys. Lett.* **2014**, *104*, 251915. doi:10.1063/1.4885215.
15. Givens, F.L.; Fredericks, G.E. Thermal Expansion of $NbSe_2$ and TaS_2. *J. Phys. Chem. Solids* **1977**, *38*, 1363–1365. doi:https://doi.org/10.1016/0022-3697(77)90008-7.
16. Bao, W.; Miao, F.; Chen, Z.; Zhang, H.; Jang, W.; Dames, C.; Lau, C.N. Controlled Ripple Texturing of Suspended Graphene and Ultrathin Graphite Membranes. *Nat. Nanotechnol.* **2009**, *4*, 562–566. doi:10.1038/nnano.2009.191.
17. Lien, H.H. Thermal Conductivity of Thin-Film Niobium Diselenide From Temperature Dependent Raman. Master Thesis, 2017.
18. Ferreiro, J.M. Experimental and Simulation Study of Electron and Phonon Properties in Crystalline Materials. PhD Thesis, University of Notre Dame, 2019.

19. Balandin, A.A.; Ghosh, S.; Bao, W.; Calizo, I.; Teweldebrhan, D.; Miao, F.; Lau, C.N. Superior Thermal Conductivity of Single-Layer Graphene. *Nano Lett.* **2008**, *8*, 902–907. doi:https://doi.org/10.1021/nl0731872.
20. Hill, H.M.; Rigosi, A.F.; Krylyuk, S.; Tian, J.; Nguyen, N.V.; Davydov, A.V.; Newell, D.B.; Walker, A.R.H. Comprehensive Optical Characterization of Atomically Thin $NbSe_2$. *Phys. Rev. B.* **2018**, *98*, 165109. doi:10.1103/PhysRevB.98.165109.
21. Weber, J.W.; Calado, V.E.; van de Sanden, M.C.M. Optical Constants of Graphene Measured by Spectroscopic Ellipsometry. *Appl. Phys. Lett.* **2010**, *97*, 091904. doi:10.1063/1.3475393.
22. Hsu, C.; Frisenda, R.; Schmidt, R.; Arora, A.; Vasconcellos, S.M.; Bratschitsch, R.; Zant, H.S.J.; Castellanos-Gomez, A. Thickness-Dependent Refractive Index of 1L, 2L, and 3L MoS_2, $MoSe_2$, WS_2, and WSe_2. *Adv. Opt. Mater.* **2019**, *7*, 1900239. doi:10.1002/adom.201900239.
23. Darvishzadeh, A.; Alharbi, N.; Mosavi, A.; Gorji, N.E. Modeling the Strain Impact on Refractive Index and Optical Transmission Rate. *Physica B: Condens. Matter* **2018**, *543*, 14–17. doi:10.1016/j.physb.2018.05.001.
24. Wang, X.; Lan, S. Optical Properties of Black Phosphorus. *Adv. Opt. Photonics* **2016**, *8*, 618–655. doi:10.1364/aop.8.000618.
25. Gil-Santos, E.; Ramos, D.; Pini, V.; Llorens, J.; Fernández-Regúlez, M.; Calleja, M.; Tamayo, J.; San Paulo, A. Optical Back-Action in Silicon Nanowire Resonators: Bolometric versus Radiation Pressure Effects. *New J. Phys.* **2013**, *15*, 035001. doi:10.1088/1367-2630/15/3/035001.
26. Boyd, E.J.; Uttamchandani, D. Measurement of the Anisotropy of Young's Modulus in Single-Crystal Silicon. *J. Microelectromech. Syst.* **2012**, *21*, 243–249. doi:10.1109/jmems.2011.2174415.
27. Shur, M. *Physics of Semiconductor Devices*, 1st ed.; Prentice Hall Series in Solid State Electronics, Prentice Hall: New Jersey, USA, 1990; p. 681.
28. Ferreiro-Vila, E.; Molina, J.; Weituschat, L.M.; Gil-Santos, E.; Postigo, P.A.; Ramos, D. Micro-Kelvin Resolution at Room Temperature Using Nanomechanical Thermometry. *ACS Omega* **2021**, *6*, 23052–23058. doi:10.1021/acsomega.1c02045.
29. Palik, E.D.; Prucha, E.J. *Handbook of Optical Constants of Solids*; Academic Press: Burlington, 1997. doi:https://doi.org/10.1016/B978-012544415-6.50000-5.
30. Suzuki, H.; Yamaguchi, N.; Izumi, H. Theoretical and Experimental Studies on the Resonance Frequencies of a Stretched Circular Plate: Application to Japanese Drum Diaphragms. *Acoust. Sci. Technol.* **2009**, *30*, 348–354. doi:10.1250/ast.30.348.
31. Lee, J.; Wang, Z.; He, K.; Shan, J.; Feng, P.X.L. High Frequency MoS_2 Nanomechanical Resonators. *ACS Nano* **2013**, *7*, 6086–6091. doi:10.1021/nn4018872.
32. Zheng, X.Q.; Lee, J.; Feng, P.X.L. Hexagonal Boron Nitride Nanomechanical Resonators with Spatially Visualized Motion. *Microsyst. Nanoeng.* **2017**, *3*, 17038. doi:10.1038/micronano.2017.38.
33. Piller, M.; Sadeghi, P.; West, R.G.; Luhmann, N.; Martini, P.; Hansen, O.; Schmid, S. Thermal Radiation Dominated Heat Transfer in Nanomechanical Silicon Nitride Drum Resonators. *Appl. Phys. Lett.* **2020**, *117*, 034101. doi:10.1063/5.0015166.
34. Murakami, Y. *Theory of Elasticity and Stress Concentration*; Wiley: Chichester, West Sussex, United Kingdom, 2017. doi:10.1002/9781119274063.
35. Papas, D.; Ou, J.Y.; Plum, E.; Zheludev, N.I. Optomechanical Metamaterial Nanobolometer. *APL Photonics* **2021**, *6*, 126110. doi:10.1063/5.0073583.
36. Blaikie, A.; Miller, D.; Aleman, B.J. A Fast and Sensitive Room-Temperature Graphene Nanomechanical Bolometer. *Nat. Commun.* **2019**, *10*, 4726. doi:10.1038/s41467-019-12562-2.
37. Siskins, M.; Lee, M.; Manas-Valero, S.; Coronado, E.; Blanter, Y.M.; van der Zant, H.S.J.; Steeneken, P.G. Magnetic and Electronic Phase Transitions Probed by Nanomechanical Resonators. *Nat. Commun.* **2020**, *11*, 2698. doi:10.1038/s41467-020-16430-2.
38. Ding, Z.; Jiang, J.W.; Pei, Q.X.; Zhang, Y.W. In-Plane and Cross-Plane Thermal Conductivities of Molybdenum Disulfide. *Nanotechnology* **2015**, *26*, 065703. doi:10.1088/0957-4484/26/6/065703.
39. Gu, X.; Li, B.; Yang, R. Layer Thickness-Dependent Phonon Properties and Thermal Conductivity of MoS_2. *J. Appl. Phys.* **2016**, *119*, 085106. doi:10.1063/1.4942827.
40. Sandell, S.; Maire, J.; Chavez-Angel, E.; Torres, C.M.S.; Kristiansen, H.; Zhang, Z.; He, J. Enhancement of Thermal Boundary Conductance of Metal-Polymer System. *Nanomaterials* **2020**, *10*, 670. doi:10.3390/nano10040670.
41. Ye, F.; Lee, J.; Feng, P.X. Electrothermally Tunable Graphene Resonators Operating at Very High Temperature up to 1200 K. *Nano Lett.* **2018**, *18*, 1678–1685. doi:10.1021/acs.nanolett.7b04685.
42. Kurek, M.; Carnoy, M.; Larsen, P.E.; Nielsen, L.H.; Hansen, O.; Rades, T.; Schmid, S.; Boisen, A. Nanomechanical Infrared Spectroscopy with Vibrating Filters for Pharmaceutical Analysis. *Angew. Chem., Int. Ed.* **2017**, *56*, 3901–3905. doi:10.1002/anie.201700052.
43. Kurek, M. Photothermal IR Spectroscopy with Perforated Membrane Micromechanical Resonators. PhD Thesis, Technical University of Denmark, 2017.
44. Barnes, J.; Stephenson, R.J.; Woodburn, C.N.; O'Shea, S.J.; Welland, M.E.; Rayment, T.; Gimzewski, J.K.; Gerber, C. A Femtojoule Calorimeter using Micromechanical Sensors. *Rev. Sci. Instrum.* **1994**, *65*, 3793–3798. doi:10.1063/1.3397320.
45. Babaei Gavan, K.; van der Drift, E.W.J.M.; Venstra, W.J.; Zuiddam, M.R.; van der Zant, H.S.J. Effect of Undercut on the Resonant Behaviour of Silicon Nitride Cantilevers. *J. Micromech. Microeng.* **2009**, *19*, 035003. doi:10.1088/0960-1317/19/3/035003.
46. Fishlock, S.J.; O'Shea, S.J.; McBride, J.W.; Chong, H.M.H.; Pu, S.H. Fabrication and Characterisation of Nanocrystalline Graphite MEMS Resonators using a Geometric Design to Control Buckling. *J. Micromech. Microeng.* **2017**, *27*, 095015. doi:10.1088/1361-6439/aa7ebb.

47. Dorgan, V.E.; Behnam, A.; Conley, H.J.; Bolotin, K.I.; Pop, E. High-field Electrical and Thermal Transport in Suspended Graphene. *Nano Lett.* **2013**, *13*, 4581–4586. doi:10.1021/nl400197w.
48. De Alba, R.; Massel, F.; Storch, I.R.; Abhilash, T.; Hui, A.; McEuen, P.L.; Craighead, H.G.; Parpia, J.M. Tunable Phonon-Cavity Coupling in Graphene Membranes. *Nat. Nanotechnol.* **2016**, *11*, 741–746. doi:10.1038/nnano.2016.86.
49. Ramos, D.; Malvar, O.; Davis, Z.J.; Tamayo, J.; Calleja, M. Nanomechanical Plasmon Spectroscopy of Single Gold Nanoparticles. *Nano Lett.* **2018**, *18*, 7165–7170. doi:10.1021/acs.nanolett.8b03236.
50. Hecht, E. *Optics*, 5th ed.; Pearson Education Limited: Essex, England, 2017; p. 67.
51. Liu, K.; Wu, J. Mechanical Properties of Two-Dimensional Materials and Heterostructures. *J. Mater. Res.* **2015**, *31*, 832–844. doi:10.1557/jmr.2015.324.
52. Metzger, C.; Favero, I.; Ortlieb, A.; Karrai, K. Optical Self Cooling of a Deformable Fabry-Perot Cavity in the Classical Limit. *Phys. Rev. B.* **2008**, *78*, 035309. doi:10.1103/PhysRevB.78.035309.
53. Baumeister, P.W. *Optical Coating Technology*; Vol. PM137, SPIE - The International Society for Optical Engineering: Bellingham, Washington USA, 2004. doi:10.1117/3.548071.
54. Byrnes, S.J. Multilayer Optical Calculations. *arXiv:1603.02720v5* **2016**.
