# Supplementary figures and images for: Photothermal Responsivity of van der Waals Material-Based Nanomechanical Resonators
Source: Nanomaterials (Basel). 2022 Aug 4;12(15):2675. doi: 10.3390/nano12152675 (PMC9370576; doi:10.3390/nano12152675)

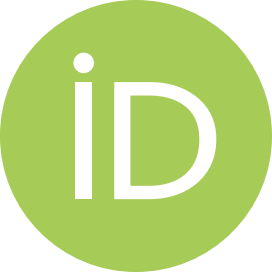

Supplement: Supplementary file 1 [file nanomaterials-12-02675-s001.zip › Definitions/logo-orcid.pdf]

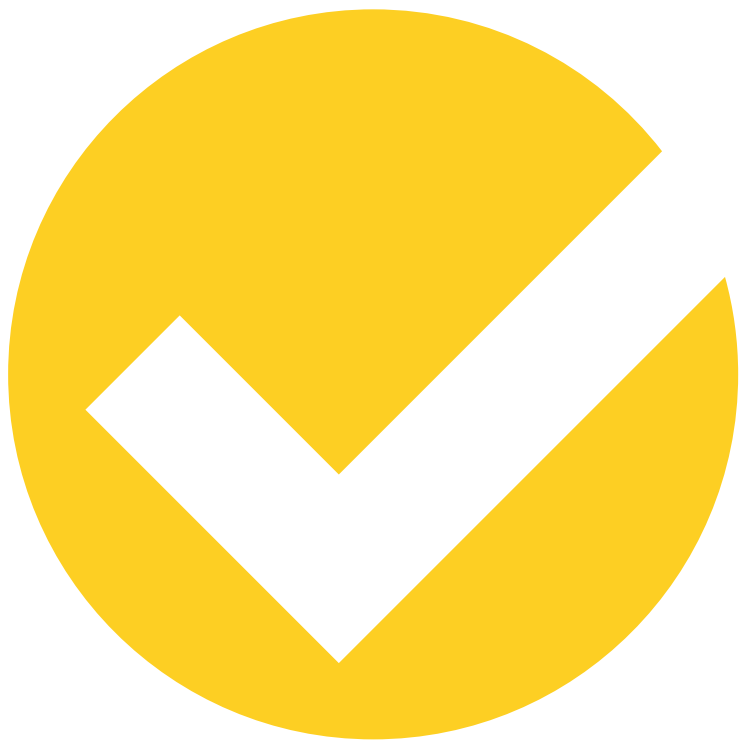

check for  
updates

Supplement: Supplementary file 1 [file nanomaterials-12-02675-s001.zip › Definitions/logo-updates.pdf]

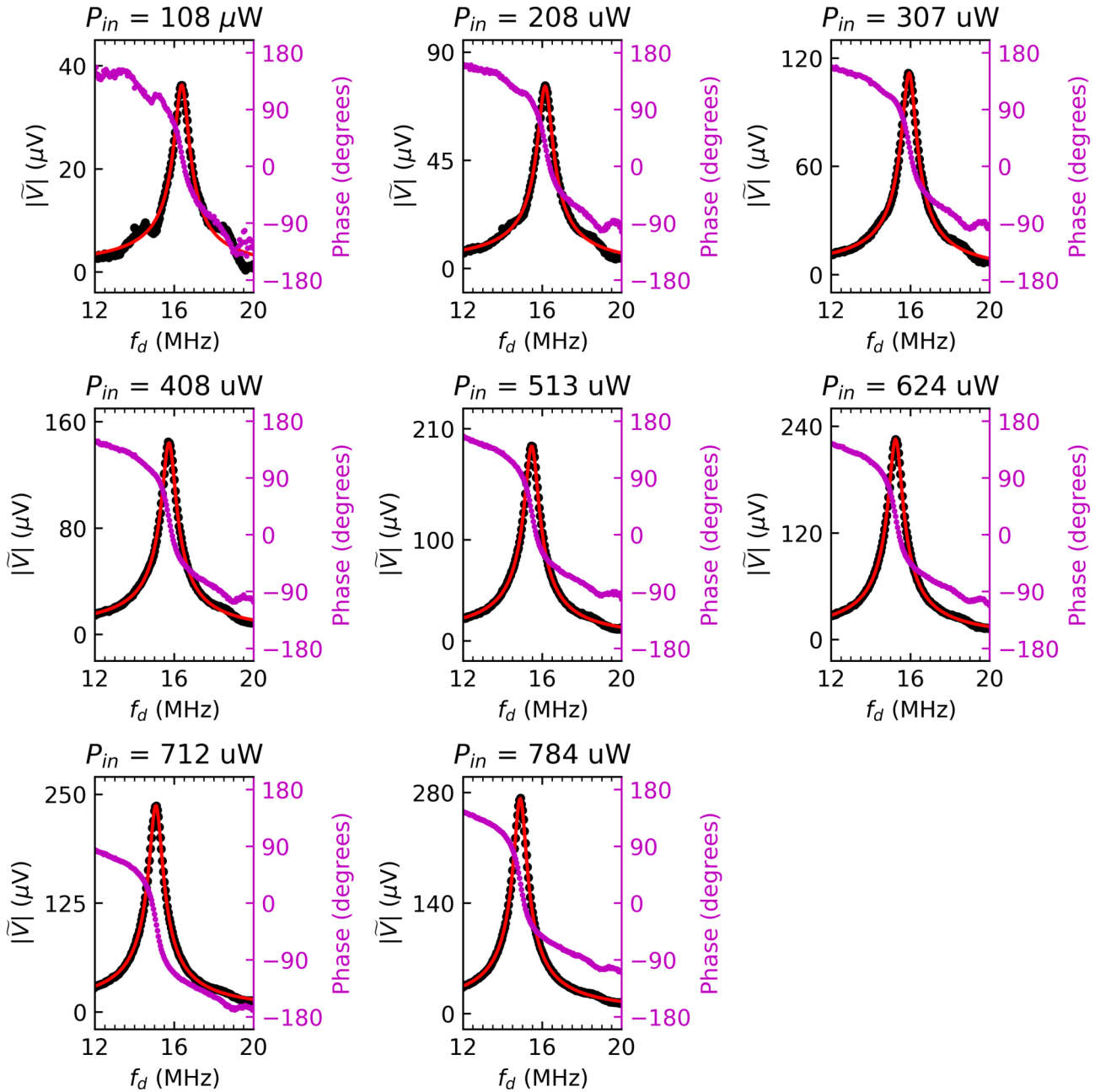

Supplement: Supplementary file 1 [file nanomaterials-12-02675-s001.zip › images_20220720/Fig_s1_raw-eps-converted-to.pdf]

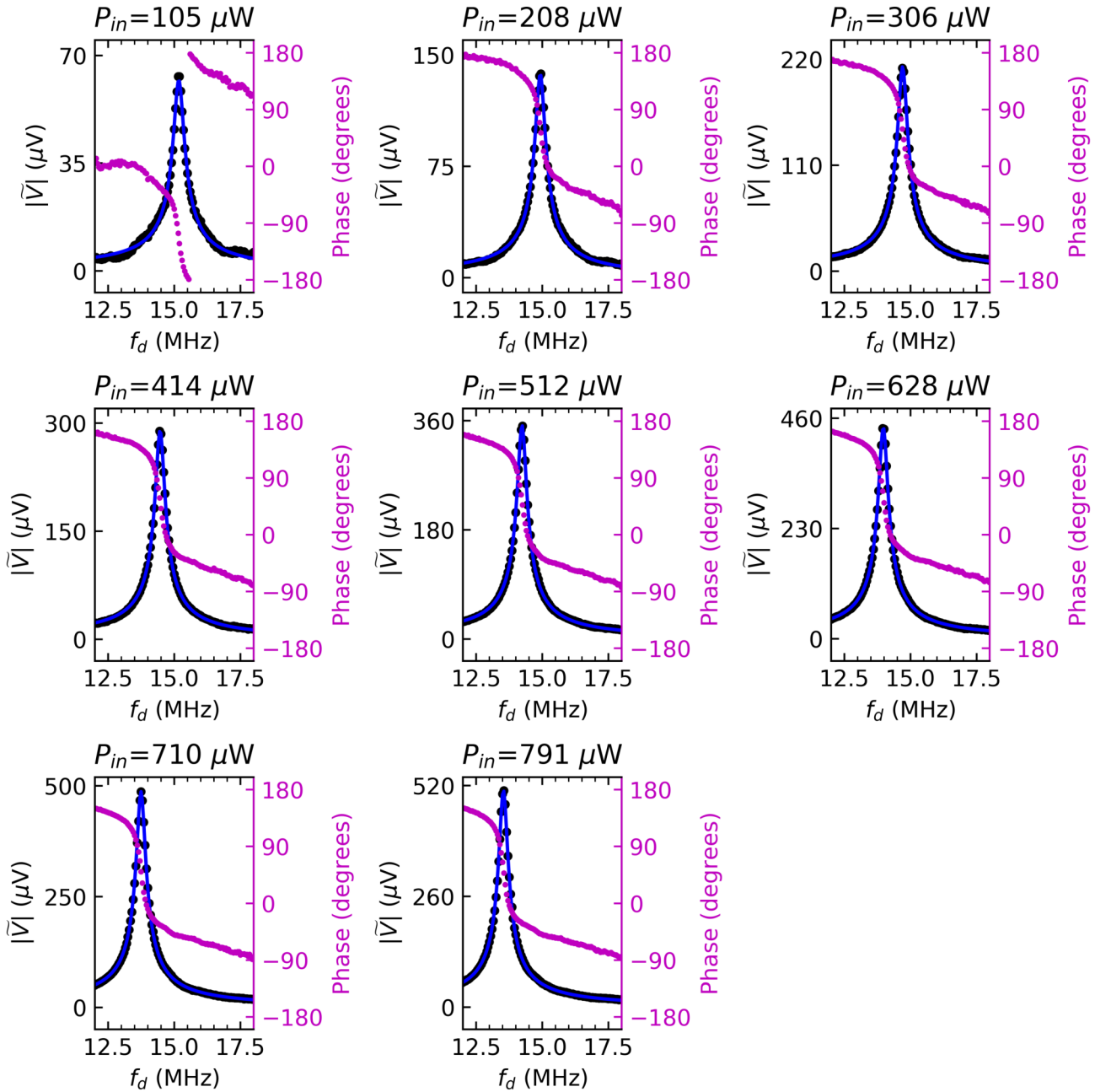

Supplement: Supplementary file 1 [file nanomaterials-12-02675-s001.zip › images_20220720/Fig_s2_raw-eps-converted-to.pdf]

(a)

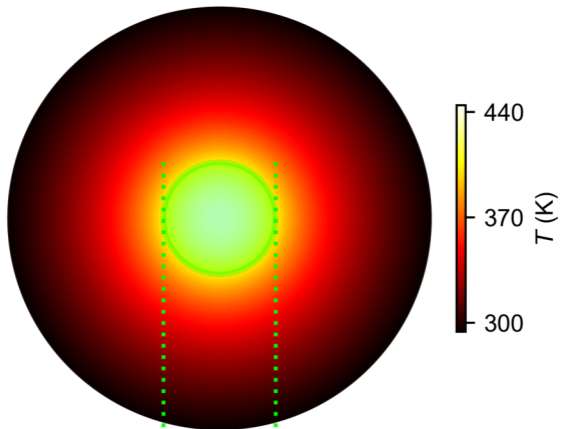

(b)

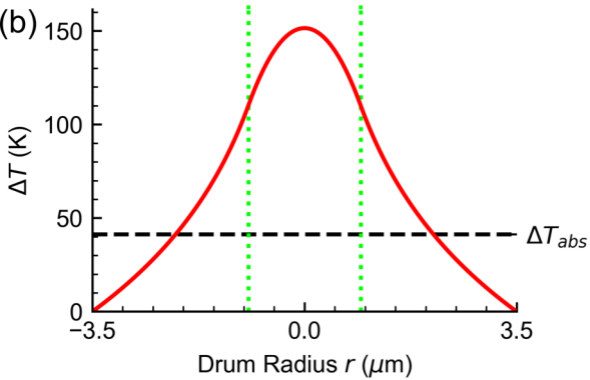

Supplement: Supplementary file 1 [file nanomaterials-12-02675-s001.zip › images_20220720/Fig_s3_drum-eps-converted-to.pdf]

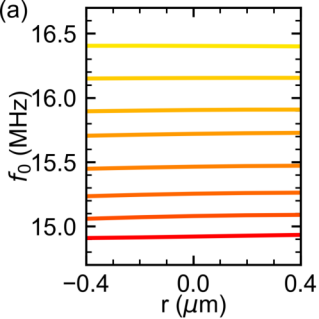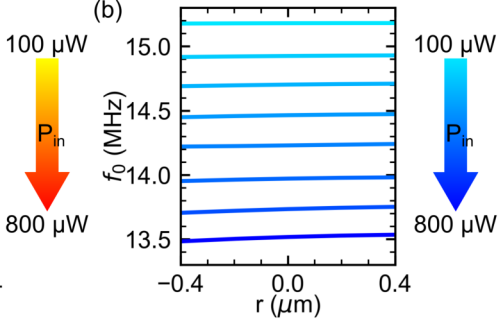

Supplement: Supplementary file 1 [file nanomaterials-12-02675-s001.zip › images_20220720/Fig_s4_align-eps-converted-to.pdf]
